# To Wear or Not to Wear: Analysis of Individuals’ Tendency to Wear Masks during the COVID-19 Pandemic in China

**DOI:** 10.3390/ijerph182111298

**Published:** 2021-10-27

**Authors:** Min Wang, Caiyue Zhao, Jing Fan

**Affiliations:** 1 Department of Business Administration, International Business School, Beijing Foreign Studies University, Beijing 100089, China; wangmin@bfsu.edu.cn (M.W.); 19021102@bfsu.edu.cn (C.Z.); 2 Department of Management Science and Engineering, Beijing Foreign Studies University, Beijing 100089, China

**Keywords:** COVID-19, mask wearing, Unified Theory of Acceptance and Use of Technology 2 (UTAUT 2), Health Belief Model, cultural difference, social influence

## Abstract

During the COVID-19 pandemic, the percentage of Chinese people wearing masks was very high, as was the acceptance and initiative toward mask wearing. This national action merits our exploration of the psychological reasons as well as the general social and environmental factors behind this behavior. In this article, we integrated the Unified Theory of Acceptance and Use of Technology 2 (UTAUT 2) as well as Health Belief Model and set up a mask acceptance model. We used a questionnaire survey and received 337 valid questionnaires. The results indicate that social influence, perceived susceptibility to COVID-19, perceived hedonic benefit (appearance enhancement), and a perceived barrier (hindrance to communication) exert significant influences on the willingness to wear masks. Meanwhile, social influence plays an intermediary role between interdependent self-construal and intention to wear a mask. We hope to reveal the micro psychological reasons for the national action and reflect on the cultural characteristics of Chinese people in the special context of the COVID-19 pandemic.

## 1. Introduction

In 2020, the coronavirus pandemic affected the entire world. Just three months after its outbreak, the pandemic had spread to more than 210 countries and regions, affecting nearly 7 billion people; by October 2021, there had been almost 238 million cases worldwide. Since the outbreak of the COVID-19 pandemic, countries have taken various responsive measures, such as mask wearing. In this complicated situation, a simple mask has become a controversial subject. To mask or not to mask? This has become a universal question.

Attitudes toward wearing masks vary widely across the United States: Washington D.C., Maine, North Carolina, etc., instituted statewide mask mandates, while there have been no mandates from governments in states such as Wyoming, South Dakota, and Utah. Topics about masks have become a political hot topic—during the U.S. presidential election in November 2020, Joe Biden and Donald Trump debated the utility of masks. Divergent opinions about mask wearing exist not only in America, but also around the globe. Starting in mid-November 2020, Korean citizens living in Seoul who did not adhere to the mask policy were fined [1]. Sweden’s government, on the other hand, stayed “calm” and steadfastly resisted mask wearing tactics [2]. In Portugal, people gathered in Lisbon to demonstrate against the imposed mask measure, calling for “freedom” and “truth” [3].

The reason why many regions in the world did not deem mask wearing as a valid measure can be attributed to a lack of medical evidence. Researchers stated that prior to the 2019 coronavirus pandemic, there was a lack of relevant data to underpin the efficacy of mask wearing in reducing the transmission of respiratory illnesses [4]. The WHO guidance on mask wearing released on January 29th, 2020, stated that “A medical mask is not required, as no evidence is available on its usefulness to protect nonsick persons. However, masks might be worn in some countries according to local cultural habits” [5]. The updated guidance on the WHO (World Health Organization) official website on 12 December 2020 said that “The use of masks alone is not sufficient to provide an adequate level of protection against COVID-19” [6].

It is worth noting that Chinese people positively and actively reacted to the appeals for mask wearing all along. China is considered to be one of the first countries to emphasize the benefit of wearing a mask among people when first reacting to the disease. In the first few days of the epidemic, a purchasing boom occurred throughout the whole country, and the percentage of Chinese people wearing masks remained high afterward. It was estimated in March 2021 that China would need 505 million face masks a day when work resumes at half capacity, which exemplifies the “enthusiasm” people have for masks. When interaction is unavoidable, such as daily shopping in the supermarket, each person was asked to wear a mask and distance themselves from others. In other words, masks have almost become a symbol of China’s campaign against the virus.

To explain the psychological reasons behind the mass “zeal” among Chinese people, we combine the UTAUT and Health Belief Model as the theoretical reference framework. Theorists have devoted little attention to issues concerning the antecedents of mask wearing. A few studies indicated that greater exposure to COVID-19-related news, perceived severity of COVID-19, negative affect (perceived stress and anxiety), and some demographics (gender) relate to mask wearing use (MacIntyre et al., 2021; Zhang et al., 2021) [7,8]. Our paper aimed to explore more culture-based predictors, including social influence and self-construal, the benefits (appearance enhancement caused by mask wearing) and the barriers (hindrance to communication).

## 2. Theoretical Model and Hypothesis Development

We developed our hypotheses under the UTAUT model and Health Belief Model; the former examines external factors, and the latter examines internal factors.

The Unified Technology Acceptance and Use of Technology model (UTAUT) was proposed in 2003 by Venkatesh et al. [9], and is an integrated theory based on eight previously developed models, such as the theory of planned behavior. In the UTAUT model, the main factors influencing users’ willingness to employ a certain technology include performance expectancy, effort expectancy, social influence and facilitating conditions. In 2012, Venkatesh et al. further improved the model, adding hedonic motivation, price value, and habit into the core variables, thus obtaining the UTAUT model [10].

Studies have shown that the UTAUT model has the highest explanatory power for final acceptance and intention toward the use of a certain technology. We found that certain factors from the model, namely, social influence, facilitating condition, and price value, are consistent with our conceived variables. Although mask wearing does not count as a kind of information technology, it is part of personal protective equipment (PPE), a simple safeguard technology functioning as a preventive measure. In previous studies, researchers used UTAUT to explain why people adopt innovative products or carry out nontraditional behaviors. The pandemic situation can be characterized as a nontraditional case, as is this national action of mask wearing. Therefore, the model applies to the research context of this paper. Additionally, UTAUT has already been widely applied in the health care industry, such as electronic health record port adoption and the acceptance of telemedicine for diabetes management [11,12]. In this article, the UTAUT model will be used to explain certain motivations for wearing masks during the pandemic.

We also used the Health Belief Model to account for other complex and subtle micro psychological factors. The Health Belief Model (HBM) was developed in the early 1950s by social psychologists from the US Public Health Service to understand the “broad failure of people to receive screening tests for disease prevention or early detection of asymptomatic diseases” [13]. The HBM consists of six constructs: perceived susceptibility, perceived severity, perceived benefits, perceived barriers, cues to action, and self-efficacy. This model has been applied in many analyses concerning health-related behavior, such as the utilization of ambulatory care services and compliance of persons with diabetes mellitus [14,15].

Among the independent variables developed in this article, the first three factors of social influence, facilitating condition and price value are derived from the UTAUT model, with self-construal serving as the antecedent of social influence. Perceived susceptibility, perceived benefit, and perceived barriers are factors included in the Health Belief Model.

### 2.1. UTAUT Model

#### 2.1.1. Social Influence and Intention to Wear Mask

According to the UTAUT model theory, social influence explains the degree of importance an individual senses that he or she should assign to the technology based on others’ opinions [9]. It has long been known that individuals adjust their thoughts to the group norm because they can acquire influential information from peers’ reactions [16]. Venkatesh and Davis differentiated between voluntary and mandatory contexts, in which social influence functions differently (Venkatesh and Davis, 2000) [17]. Previous empirical research shows that the opinions of significant others, such as friends, peers, and coworkers, positively influence the intentions of diabetic patients to use telemedicine and drivers’ tendency to apply wearable health monitoring technology [12,18].

From the very start of the pandemic, Chinese disease control authorities and various news media have stressed the importance of masks. The leader of the expert group of the China National Health Commission, Nanshan Zhong, who has a high reputation and is influential in the medical world and in the eyes of the public, emphasized the importance of mask wearing on different occasions. Instructions and news about mask wearing is also abundant on official media and social platforms. There are signs reminding people in stores, supermarkets, and metro stations, thus creating intense pressure from the surroundings for citizens to consciously wear masks, not just for themselves but also for other people. Therefore, in this large-scale campaign, mask wearing concerns not only an altruistic intention to slow down the transmission speed, but also the social pressure that people not wearing masks are irresponsible both for others and for themselves.

Based on a survey among college students, Gette et al. found that participants’ perceived norms for mask wearing significantly predicted their indoor and outdoor mask wearing behaviors [19]. The effect of social influence may be more pronounced in China. According to the cultural dimension theory developed by Hofstede, China is a country with a higher power distance. It has been clearly shown that the information people receive from professional and official institutions can provide the public with more positive and confident attitudes toward COVID-19 [20]. The government has the authority to enact mandatory measures to wear face masks. Other social institutions echoed the call from the government and advertised the importance of masks with their social credibility. These influential factors added together, and people correspondingly reacted to national appeals according to their own will.

Therefore, based on the conditions above, the following hypothesis can be proposed:

**Hypothesis** **1** **(H1).** *Social influence is positively related to people’s willingness to wear masks.*

#### 2.1.2. Facilitating Condition

Based on the UTAUT model, facilitating conditions will also have an effect on behavioral intention. The definition of facilitating conditions is the degree to which individuals believe that the utilization of a system is supported by certain organizational and technical infrastructure [9]. Some previous studies have indicated that the facilitating conditions can predict diabetes patients’ telemedicine use intention [11] and individuals’ intention to use wearable health monitoring technology [18].

In this study, we define the facilitating conditions as individuals’ convenience of purchasing masks. In the first month after the outbreak, China experienced a severe mask shortage. Obtaining masks in nearby drugstores and online shopping websites was difficult, which are common facilitating places for mask purchasing. Although the Chinese public is keenly aware of the importance of masks, they are still passively affected by the inconvenience of obtaining masks. After production capacity resumed, the output of masks increased greatly, and the difficulty of buying masks was lessened, which increased individuals’ willingness to buy and wear masks. Therefore, this study proposes the following hypothesis:

**Hypothesis** **2** **(H2).** *The purchasing availability of facial masks is positively related to individuals’ willingness to wear masks.*

#### 2.1.3. Price Value

Price value in the model is defined as individuals’ cognitive tradeoff between perceived benefits and monetary cost for applying them. It is proposed that individuals’ use of technology would be significantly influenced by the cost and pricing structure. When the benefits of applying a certain technology are recognized to be greater than the cost, the price value has a positive influence on intention [10]. Some existing studies validated the positive effect of PV on individuals’ intention to use wearable health monitoring technology (Binyamin and Hoque, 2020) [18]. In this paper, it is assumed that price value will also be positively related to people’s willingness to buy and use masks, because masks are also deemed preventive health appliances.

The mask shortage was accompanied by an abnormally high price, which partly also resulted from the increase in price. In this context, the benefits are unable to outweigh the monetary cost, because the raw material cost of masks is very low and a great amount of extra money had to be spent on masks, thus curbing people’s intention to purchase. Instead, they may use alternative methods, such as staying at home and adhering to stricter social distancing rules. Therefore, based on the statement above, the following hypothesis is postulated:

**Hypothesis** **3** **(H3).** *The price value of mask wearing is positively related to individuals’ willingness to wear masks.*

### 2.2. Self-Construal Theory—Self-Construal and Social Influence

Although conformity is a universal human activity, the degree to which people are affected by others and comply with certain social norms is different. Social influence alone cannot sufficiently interpret high acceptance of masks among the Chinese population, because individuals are the constituent unit of the collective. Therefore, in this paper, an individual-level factor—self-construal—is placed ahead of social influence—an interpersonal-level variable.

Singelis (1994) [21], who proposed self-construal theory, defined self-construal as a composition of thoughts, feelings, and actions regarding one’s relationship with others as well as the disparate self from others. Additionally, he differentiated the means of gaining self-esteem between two types of self-construal: for independent self-construal persons, they obtain self-esteem through proper and free expression of self, while for interdependent persons, their source of gaining self-esteem lies in harmonious interpersonal relationships and timely adjustment to the situation. According to Markus and Kitayama (1991) [22], a significant difference between Easterners and Westerners was the extent to which the self was defined in terms of relationships with others. In regions such as Asia, Southern Europe, and South America, where collectivist culture is advocated, the individual’s definition of self is mainly based on his or her relationship with others and status, as well as identity in the group. People tend to build codependent selves, the basic goal of which is to maintain interpersonal relationships. However, whether and how this goal can be achieved depend largely on the situation, especially the interacting objects in the situation. In many cases, the expression of individual representations needs to be suppressed or controlled to maintain interpersonal relationships.

In contrast, people in Western countries such as the United States and Australia believe in individualism and pursue independence. Individuals tend to see themselves as a unique and stable system, a dynamic center that can integrate individual consciousness, emotion, judgment, and behavior. This center is opposed not only to the systems of other individuals but also to the social and natural context in which they live. Under these conditions, independent self-construal is formed.

Concerning wearing a mask, Western people may see face masks as a restriction of their freedom, while Chinese people tend to take the group’s well-being into consideration and conform to collective behavior. In other words, they wear a mask not only for their well-being, but also for the group’s benefit and due to the pressure to behave the same. For the public, wearing masks is more of an attitude, the attitude to unite and fight against the virus.

**Hypothesis** **4a** **(H4a).** *People’s interdependent self-construal (ISC) is positively related to their willingness to wear masks.*

**Hypothesis** **4b** **(H4b).** *Social influence plays a mediating role between ISCs and people’s willingness to wear masks.*

### 2.3. Health Belief Model Theory

#### 2.3.1. Perceived Benefits

##### Medical Function

Originating from the Health Belief Model, the construct of perceived benefits refers to a person’s belief in potential beneficial aspects of a health-related action to reduce the threat or seriousness of illness or disease. Perceived susceptibility can provide a driving force to action, but alone, it cannot determine the course of action. Certain benefits must be perceived to finally take an action available [23]. In other words, unless a certain health measure is considered feasible and effective, an individual will not accept the recommended health action. A study on delaying looking for medical confirmation of cancer showed that a lack of predicted effective access prevents individuals from seeking diagnoses for cancer symptoms, despite the strong feeling of susceptibility to the most feared illness [24].

In this specific COVID-19 situation, an increasing number of people have realized the importance of self-care measures. Many people believed that mask wearing helped to efficiently control the transmission of SARS in China in 2003. Now, with increasing medical evidence and appeals from authoritative institutes, some people acknowledged the medical function of the mask. With the perceived positive outcome of wearing masks, people are more willing to put them on. Accordingly, we propose the following hypothesis:

**Hypothesis** **5a** **(H5a).** *The perceived medical function of masks is positively related to individuals’ willingness to wear masks.*

##### Hedonic Benefit: Appearance-Enhancement

Apart from health-related benefits, mask wearing could bring another positive outcome: enhancement of appearance. Although the original intention of wearing masks is to prevent people from becoming infected, it was soon discovered that wearing masks appeared to be capable of increasing facial attractiveness, which even sparked plenty of discussions on the Internet.

As reported in a Japanese variety show, the staff conducted street interviews to prove this point: they asked female interviewees to remove their masks in front of the camera and found they were significantly more confident when wearing masks than without masks [25]. Gestalt psychology proposes that the visual image is perceived first as a unified whole and then as a part, which helps in accounting for the effect that mask wearing has on improving one’s overall facial appearance. The part covered by the mask will be completed instinctively by individuals, generally beneficially. Additionally, blemishes on the cheeks and chin disappear with the mask on.

Wearing a mask will not only make faces look smaller and more attractive, but it will also promote individuals’ imagination about the outlook of another person because half of the face is hidden behind the mask. Given the prevailing “facial attractiveness economy”, it seems that this improved function of wearing masks does contribute to people’s tendency to wear masks. The following hypothesis is thus developed:

**Hypothesis** **5b** **(H5b).** *Perceived hedonic function of masks to enhance one’s appearance is positively related to individuals’ willingness to wear masks.*

#### 2.3.2. Perceived Barriers

According to the Health Belief Model, perceived benefits and barriers work together to determine the occurrence of a given action [23]. In some cases, perceived barriers offset parts of benefits, and in others, the benefits outweigh barriers. Although it has been proven that mask wearing contributes to slowing down the transmission of the disease, some disadvantageous factors, such as unreasonable prices and side effects such as breathing difficulties as well as hindrances to communication, cannot be neglected. As China is considered a high context culture, people tend to understand each other from the facial expressions. In that case, long-term mask wearing would serve as an obstacle to interpersonal communication. Therefore, in everyday situations, a mask blocks signals or hidden thoughts, because the eyes need to work together with the nose and mouth to make appropriate expressions. This leads to the following hypothesis:

**Hypothesis** **6** **(H6).** *Perceived barriers to masks are negatively related to individuals’ willingness to wear masks.*

#### 2.3.3. Perceived Susceptibility

According to the Health Belief Model [13], perceived susceptibility describes an individual’s perception of the risk of having a certain illness. There is wide variation in a person’s awareness of personal vulnerability to contracting a disease. Some are convinced that they will acquire the disease, while some deny that the risk of contracting the condition exists. In a more moderate situation, people acknowledge the mathematical possibilities of acquiring the illness. It is easy to understand that when a person finds himself or herself highly exposed to potential illness and disease, he or she would be more urgent and willing to take preventive actions. Some studies have reflected a strong correlation between perceived susceptibility to HIV and positive behavior change [13,26,27]. Additionally, studies have shown that clients who are motivated by perceived susceptibility to dental disease seek dental care [28]. When faced with a large-scale infectious disease such as COVID-19, people naturally take preventive measures driven by fear of contracting the disease. Therefore, to test the potential relationship between perceived susceptibility and preventive health-related action (namely, mask wearing action), COVID-19 concern data were collected in this study, which refers to the degree to which individuals worry they will be infected by the virus. The following hypothesis is proposed:

**Hypothesis** **7** **(H7).** *Concern about contracting COVID-19 is positively related to individuals’ willingness to wear masks.*

## 3. Methodology

### 3.1. Sample and Procedure

The data collection procedure was completed in May 2020. During this month, mainland China reported 1 new death and 120 confirmed cases. For the first time since the outbreak, the average number of new cases has dropped to less than 10, decreasing from thousands per day at the peak. As a result, the public came to believe that China has managed to control the pandemic. The anxiety and fear triggered by COVID-19 largely lessened. Meanwhile, the temperature was rising in most provinces, thus making mask wearing more uncomfortable. The overwhelming majority of Chinese people strictly carried out the mask wearing policy from January to April. However, in May, some people stopped wearing masks. Therefore, it was a good point in time for researchers to investigate the underlying reasons why people wear masks.

We collected data by sending out an online questionnaire. The project was introduced as a university research project. Approximately 360 people were enrolled in the study, and each received RMB 20 as a participation fee. We excluded the responses that were finished in less than 120 s or those “straight liners”, i.e., those who chose the same answers over 6 questions in a row. After the cleaning process, the sample consisted of 337 people from 26 provinces. Among the participants, 56% were men and females took up 44% of the cohort, and about 80% were married.

### 3.2. Measures

The survey consisted of two sections. The first section included demographic variables (gender, age, residence, education, health, allergy history, etc.). The second section contained the main constructs in the model. Most of the questions in the second section were measured using a five-point Likert scale ranging from “strongly disagree” (1) to “strongly agree” (5).

Interdependent self-construal (ISC): ISC was measured with the interdependent subscale of the Self-Construal Scale (SCS) developed by Singelis (1994) [21]. We used the Chinese version proposed by Wang et al. (2008) [29]. Using a 5-point rating scale, respondents indicated how much they see themselves as connected, similar, and interdependent with others (e.g., “I often have the feeling that my relationships with others are more important than my accomplishments.”).

Social influence: Social influence was measured with the scale proposed by Venkatesh et al. (2012) [10] with minor modifications. Based on the original 3-item scale (e.g., “People who are important to me think that I should use mobile internet”), we specified the subjects who influenced individuals. Sample items are “local government think that citizens should continue wearing masks” and “my friends think that we should continue wearing masks”.

Facilitating condition: Venkatesh et al. (2012) used four items to measure facilitating conditions (e.g., “I have the resources necessary to use mobile Internet”; “I have the knowledge necessary to use mobile Internet”). Since mask-wearing is easily learned, the main obstacle is whether people can buy masks. We modified the first item in Venkatesh et al.’s (2012) scale: “I can easily buy masks I need”.

Price value: Similarly, we used one item from Venkatesh et al.’s (2012) [10] 3-item scale with minor modifications. The question was whether the participant can buy masks at a reasonable price.

Perceived benefits (medical function of mask): So far, evidence regarding the benefits of masks during the pandemic is lacking or mixed. Nevertheless, the WHO has updated relevant guidance and recommendations. In this paper, we focus on people’s views on the benefits of mask-wearing. Participants were asked to evaluate the extent to which they thought wearing masks would help to protect them from COVID-19, with the percentage scale ranging from 100% to 0%.

Perceived benefits (appearance enhancement): To our knowledge, there is no scale measuring appearance enhancement. Most of the research on appearance may use the Body Dissatisfaction Scale or Appearance Anxiety Scale. Research that includes appearance enhancement often manipulates the variable by making the participants wear cosmetics (DelPriore et al., 2018) [30]. Therefore, we developed a 3-item scale after consultation with 2 psychologists. A sample item is “Wearing masks improves my appearance”.

Perceived barriers (hindrance to communication): Perceived barriers were conceptualized as a hindrance to communication, so we adopted the Perceived Anonymity Measurement Instrument developed by Hite et al. (2014) [31] and made some adjustments to reflect the current situation. A sample item is “I am easily identified by others when I wear a mask (reverse coded)”.

Perceived susceptibility: We asked the participants a single question, “Are you worried about contracting COVID-19?”. Participants indicated the extent with the 4-point scale ranging from (1) “not so worried” to (4) “extremely worried”.

Willingness to wear a mask: Participants were asked to indicate whether they will continue wearing masks in the next few months on a 6-point Likert scale ranging from “rarely” to “definitely yes”.

### 3.3. Analysis

Two analysis software programs were used to complete this study: SmartPLS 3.0 (SmartPLS GmbH, Bönningstedt, Germany) and SPSS 26 (IBM, New York, NY, USA). The data analysis consisted of three parts. The demographic description and regression analysis were carried out with SPSS 26. SmartPLS was employed to conduct conformation confirmatory factor analysis and structural equation modeling analysis to evaluate the research model and test the hypotheses.

## 4. Data Analysis and Results

### 4.1. Reliability

In this study, the reliability of each scale was evaluated by testing internal consistency using SPSS 26 software.

The reliability coefficient values of the measurement scale of social influence, appearance enhancement, and self-construal are 0.820, 0.839, and 0.818, respectively, all exceeding 0.8, indicating a sufficient reliability level.

However, it should also be pointed out that the reliability of the perceived barriers (hindrance to communication) is 0.5, largely because this questionnaire had only two items.

### 4.2. Validity

To test the validity of the variables, this study calculated the factor loading of each indicator: the extracted value of the average variance of each latent variable (AVE) and the combined reliability (CR).

#### 4.2.1. Exploratory Factor Analysis (EFA)

Because this study involved some original new questionnaires and items, exploratory factor analysis was also conducted.

The KMO values of social influence, appearance enhancement and self-construal were 0.766, 0.659 and 0.859, respectively, all suitable for the next analytical step: confirmatory factor analysis.

The KMO values of purchase availability and perceived barriers were both 0.5. However, as stated above, this can be caused by the number of items in both questionnaires. Each of the two questionnaires only contains two items. Still, the regression results were ideal, so the two variables were retained.

#### 4.2.2. Confirmatory Factor Analysis (CFA): Convergent Validity and Model Fit

SmartPLS 3.0 was applied to conduct CFA, including convergent validity, discriminant validity, and overall model fit analysis. To test the validity of the variable measurement, this study calculated the factor loading, average variance extracted (AVE), and combined reliability (CR) of each indicator. The results show the following:

1. Factor loadings of most measurement indexes were greater than 0.6.

2. The average variance extracted (AVE) values of seven out of eight latent variables were greater than 0.5, which conforms to the discriminant standard proposed by Hair et al. (2019) [32]. The composite reliability of each latent variable reached 0.8. These results show that all the latent variables in this study reached the recommended standard for convergent validity. The overall model fit was measured with the NFI value (0.704). The test results of the above-described indexes are summarized in Table 1.

#### 4.2.3. Discriminant Validity

According to Fornell and Larcker (1981) [33], the average variance extract of each variant must be greater than the square of the correlation coefficient between the paired variants to establish discriminant validity. Hence, if it can be proven that the minimum AVE between all constructs is greater than the square value of the maximum value in the correlation coefficient matrix, then there is good discriminant validity. As seen from the chart below, each AVE value on the diagonal line is greater than the correlation value in the same column to which it belongs, and so the standard for discriminant validity was reached.

### 4.3. Correlation Analysis

The mean, standard deviation, and correlation coefficient of each variable are presented in Table 2. There was a significant positive correlation between appearance enhancement (r = 0.162, *p* < 0.05), social influence (r = 0.440, *p* < 0.05), interdependent social construal (r = 0.185, *p* < 0.05), and intention to wear the mask, while the correlation between perceived susceptibility and future mask prediction was negative. The other three independent variables, namely, facilitating condition, price value, and medical function, were not significantly correlated with the dependent variable. However, the effect of independent variables on dependent variables requires more specific regression tests.

### 4.4. Regression Results

**Figure 1:**
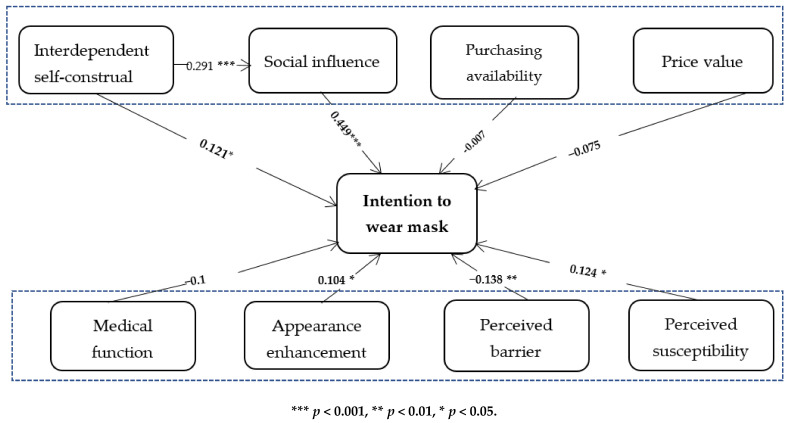
Overall regression results.

In this study, SPSS 26 was used for regression analysis to test the hypotheses. First, the factors in the UTAUT model were tested. After entering control variables, factors from the UTAUT model were set in the regression process. It can be seen from the corresponding data results in Table 3 that both interdependent self-construal (model 2, β = 0.121, *p* < 0.05) and social influence (model 2, β = 0.449, *p* < 0.001) have significant positive correlations with the dependent variable. These statements support hypotheses 1 and 4a. Because self-construal was hypothesized as the antecedent of the interdependent variable social influence, we created an additional model to test out the mediating effect of social influence, and the plug-in application process for SPSS was applied (see Table 4). The bootstrap method was used to test the mediation effect, and a judgment was made according to whether the interval (Bootllci, Bootulci) contained 0. In this paper, the minimum standard for significance was set at 0.05. Based on the data results from Table 3, it is already proven that interdependent self-construal is positively related to the dependent variable (model 2, β = 0.122, *p* < 0.05). Additionally, as can be seen from Table 4 model 1, interdependent self-construal has a significant influence on social influence. When self-construal and social influence are put into the process together, social influence significantly related to intention to wear a mask (model 2, β = 0.431, 95% CI (0.742, 1.20)), but interdependent self-construal no longer retains its positive effect on the dependent variable (mode 2, β = 0.068, 95%CI (−0.115, 0.561)), which indicates the strong mediating effect social influence has. Thus, Hypothesis 4b is supported. The overall visual regression results are shown in Figure 1, Table 3 and Table 4.

However, the results show that the correlations between the facilitating condition (β = −0.007, *p* = 0.615), price value (β = −0.075, *p* = 0.193), and dependent variable were not significant.

For the factors from the Health Belief Model, the results in the table indicate that there were significant correlations between appearance enhancement (β = 0.104, *p* < 0.05), perceived barriers (β = −0.138, *p* < 0.05), and perceived susceptibility (β = 0.124, *p* < 0.05). Therefore, hypotheses 5b, 6 and 7 were supported. Interestingly, the medical function data (β = −0.1, *p* < 0.1) showed that there was no significant correlation between this variable and the dependent variable, which will be discussed later.

## 5. Discussion

This study aims to explore and confirm psychological reasons for the mass mask wearing action carried out in China. Adopting factors from the UTAUT model and the Health Belief Model, this thesis explored eight potential determinants for the explanation of future mask wearing prediction, including social influence, facilitating condition, price value, perceived benefit (medical function of mask and appearance enhancement), perceived barriers and perceived susceptibility.

The data analysis results illustrate that social influence, interdependent self-construal, perceived benefit (appearance enhancement), perceived barriers, and perceived susceptibility have a positive impact on the dependent variable. Additionally, social influence plays an intermediate role between interdependent self-construal and future mask prediction. As seen from the results, among all the factors, social influence strongly predicts individuals’ intention to wear masks. This conforms with the general psychological perception people have of Chinese culture. Living in a collectivist culture, people in China and many other Asian countries share a more compact living space and closer social distance. Opinions from others strongly influence individuals’ opinions about themselves and their surroundings.

Social norms and others’ opinions also shape the formation process of one’s self-awareness, which explains the positive influence self-construal has on the dependent variable. Under the circumstances that COVID-19 is highly contagious and that China has the highest population density, home quarantine and mask wearing are well-accepted measures and are expected to play a very important role in the campaign. Apart from the practical effect, social norms and conventional thinking also urge people to keep masks on: when someone appears in a public place without a mask on and even expresses aloofness toward others’ well-being concerning the virus, this behavior may be reported in the news.

Perceived susceptibility also contributes to the willingness to wear a mask. With the rapid speed of transmission and mass media coverage about the disease, individuals would have easy access to information about the COVID-19 pandemic and consciously wear a mask for health purposes.

While fighting the virus and preventing it from spreading to others, masks provide an additional advantage: appearance enhancement and hedonic motivation. Masks create the effect of making the face smaller and more attractive. This fits the currently popular “facial attractiveness economy” trend.

Despite perceived benefit, individuals weigh the benefits against the barriers that a certain conscious health-related action has. In this case, the obstacle individuals encounter is the hindrance to communicating with others. Nonverbal cues are a large part of daily communication, which is particularly true with children and individuals with hearing impairment, and especially true in China, a nation with a high-context culture. People need the assistance of facial expressions to judge one’s state of mind and communicate properly. Therefore, the results show a negative statistical relationship between hindrance to communication and the prediction for future mask wearing.

On the other hand, the results demonstrate that facilitating conditions, price value, and medical function are not key determinants for the dependent variable. This is attributable to the time when data were collected. This dataset was collected in May 2020, at which time the shortage of masks was solved with the resumption of work. Therefore, it was no longer an urgent problem for people to buy masks through reasonable sources with fair prices. As the temperature rose, the situation of the pandemic also improved. Therefore, medical function is not a crucial determinant for the dependent variable.

## 6. Conclusions

This study applied the UTAUT model and the Health Belief Model as the theoretical framework to explain the factors predicting individuals’ intention to purchase and wear face masks during the pandemic situation. Social influence, perceived benefits (appearance enhancement), perceived barriers (hindrance to communication), and perceived susceptibility were significant predictors of the intention to wear masks, while facilitating conditions, price value, and perceived benefits (medical function of masks) were not significant.

### 6.1. Theoretical and Practical Significance

As indicated previously, Chinese people showed a strong and consistent tendency to wear masks from the start of the pandemic onwards. This paper explores potential factors accounting for the high acceptance rate and indicates that social influence, interdependent self-construal, appearance enhancement and perceived barriers (hindrance to communication) are significant predictors.

Furthermore, this study integrates concepts from UTAUT and the Health Belief Model to develop theoretical frameworks, providing support for both theories. The constructs chosen from the UTAUT model precisely align with the key components influencing individuals’ mindset when considering wearing masks. Social influence is a very strong factor affecting the dependent variable. Individuals naturally take others’ actions, statements and opinions into consideration when trying to blend into society. To some extent, this mask wearing measure shifts the focus from self-protection to altruism. Additionally, we conceptualized perceived benefits (appearance enhancement) and perceived barriers (hindrance to communication) from HBM, shedding some light on model development.

Finally, this study contributes to knowledge in cross-cultural fields. Chinese people are prone to possessing interdependent self-construal, value interpersonal communication and adhere rigidly to social influence, leading to high acceptance of masks, which advances the understanding of culture’s influence on public health.

To develop “nudges” to encourage healthy behaviors, policymakers and practitioners in public health should be careful with these characteristics. For example, if the public service advertisement emphasizes that “wearing a mask makes you more attractive”, people may realize the added benefits of masks and, therefore, show a higher acceptance. In addition, masks manufacturers could create more stylish patterns and types of masks, so that people can explore aesthetic value in masks. Additionally, manufacturers may develop transparent plastic face masks, thus people would not worry about the weakened connections to others caused by wearing masks. Other than the government, medical institutions can also release updated medical evidence regarding the effectiveness of masks and encourage citizens to wear masks when necessary.

### 6.2. Limitations and Future Research

There are some limitations to this study. The first issue is that the sample, containing 330 participants, is relatively small. As we were uncertain as to how the pandemic would play out, we collected the data in three days, which greatly limited the sample size. Moreover, as the paper used PLS-SEM as the modeling method, we followed the “10-times rule” to determine the sample size—there are 27 items included in the model, and therefore, 270 persons would be acceptable. To enhance the validity, we expanded the required sample size to 360 people, and ended up with 337 valid samples. In future studies, the database could be expanded to add to the validity and reliability of this model. Second, since mask-wearing is not an established topic, we developed several context-specific scales for the paper, including facilitating conditions, price value, and perceived benefits. As a result, the reliability for some constructs is not satisfying enough. Future research should develop and validate the scales to enhance the statistical power. Third, the research only included disposable surgical masks, which reduced the generalizability of the results. Future studies should take N95 respirators and other types of masks into consideration. Finally, as the study focused on one country, the generalizability of the results is greatly limited. The mechanisms underlying mask acceptance or rejection in other countries still need to be explored.

An important avenue for further research would be to further analyze the potential predictors as well as the mechanisms at the individual level. For example, does gender or social status matter? Men may be less willing to wear face masks than women because of the potential perception of being weak. Alternatively, individuals with higher status may feel more powerful and, thus, ignore social norms, and so they may tend to refuse masks. Personality traits (e.g., conscientiousness) may exert influence in the decision to wear or not wear masks. Further research is needed to elucidate these predictors.

## Figures and Tables

**Table 1:** Convergent validity, reliability, VIF and NFI results.

Construct	Factor Loading	AVE	CR	Cronbach’s α	NFI	VIF
ISC	0.668	0.337	0.85	0.809	0.704	1.608
	0.666					1.545
	0.676					1.614
	0.619					1.541
	0.654					1.534
	0.603					1.53
	0.646					1.586
	0.294					1.096
	0.566					1.461
	0.68					1.623
	0.418					1.211
	0.24					1.259
Social influence	0.616	0.526	0.868	0.816		1.394
	0.665					1.547
	0.621					1.275
	0.778					2.125
	0.814					2.536
	0.825					2.121
Appearance enhancement	0.928	0.757	0.902	0.837		3.159
	0.928					3.281
	0.74					1.466
Perceived barriers (hindrance to communication)	0.845	0.664	0.798	0.496		1.122
	0.784					1.122

**Table 2:** Mean, standard deviation and correlation of the main variables.

	Variable	Mean	SD	1	2	3	4	5	6	7	8
1	ISC	3.795	0.516	0.58							
2	Social influence	3.972	0.750	0.315 **	0.725						
3	Facilitating condition	4.340	0.932	0.295 **	0.164 **	1					
4	Price value	4.260	0.916	0.362 **	0.200 **	0.622 **	1				
5	Medical function	8.660	2.057	0.365 **	0.210 **	0.167 **	0.248 **	1			
6	Appearance-enhancement	3.036	1.085	0.128 *	0.121 *	0.022	0.075	0.010	0.87		
7	Perceived barriers	3.294	0.955	0.116 *	0.069	0.083	0.055	−0.015	−0.049	0.814	
8	Perceived susceptibility	3.420	0.494	0.028	−0.016	−0.152	−0.092	−0.050	−0.128	−0.051	1
9	Intention to wear mask	3.982	1.685	0.185 **	0.440 **	0.004	0.009	0.021	0.162 **	−0.111 *	0.152 **

*** *p* < 0.001, ** *p* < 0.01, * *p* < 0.05.

**Table 3:** Results of hierarchical regression analysis.

Dependent Variable	Intention to Wear Masks	
	Model 1	Model 2
Control variable		
Hubei province	−0.112 *	−0.053
Risk	0.038	−0.02
Prediction ^1^	0.025	0.034
Gender	0.02	0.053
Age	−0.222 *	−0.227 **
Marriage status	0.38	−0.027
With children	0.132	0.119
Health	−0.056	0.001
Allergy	−0.056	−0.068
Education	−0.068	−0.082
Income	−0.027	0.007
UTAUT model		
Interdependent self-construal		0.121 *
Social influence		0.449 ***
Facilitating condition		−0.007
Price value		−0.075
Health Belief Model		
Medical function		−0.1
Appearance-enhancement		0.104 *
Perceived barriers		−0.138 **
Perceived susceptibility		0.124 *
*R* ^2^	0.059	0.315
Δ*R*^2^	0.059	0.256
*F*	1.863	7.680 ***

^1^ Prediction here refers to the individuals’ prediction of the likelihood that the COVID-19 pandemic would once again break out in the winter of 2020. *** *p* < 0.001, ** *p* < 0.01, * *p* < 0.05.

**Table 4:** Mediation effect testing.

Dependent Variable	Social Influence	Intention to Wear Masks	
	Model 1	Model 2	
***Control variable***			
Hubei	−0.088	−0.073	
Risk	0.022	0.037	
Prediction	0.053	0.03	
Gender	−0.036	0.06	
Age	0.043	−0.241	
Marriage	0.056	−0.021	
With children	0.02	0.119	
Health	−0.07	−0.001	
Allergy	0.029	−0.05	
Education	−0.015	−0.088	
Income	−0.001	−0.016	
Independent variable	*p value* and Bootllci, Bootulci		
ISC	0.291 ***	0.068 (−0.115, 0.561)	
	(0.267, 0.580)		
Mediating variable	*p value* and Bootllci, Bootulci		
Social influence		0.431 *** (0.742, 1.20)	
*R* ^2^	0.122		0.256

*** *p* < 0.001.

## Data Availability

The full dataset presented in this study is available on request from the corresponding author, Jing Fan.
